# The sigmoidea ima artery: A player in colonic ischemia?

**DOI:** 10.1002/ca.23552

**Published:** 2020-01-10

**Authors:** Christoph Landen, Manuel Dreu, Andreas Weiglein

**Affiliations:** ^1^ Department of Macroscopic and Clinical Anatomy Medical University of Graz Graz Austria

**Keywords:** colonic ischemia, inferior mesenteric artery, marginal artery, sigmoidea ima artery, Sudeck's critical point

## Abstract

**Introduction:**

The sigmoidea ima artery is defined as the lowest sigmoid artery, which forms the distal end of the marginal artery by linking with the superior rectal artery. It supplies the rectosigmoid junction, which is a critical area for ischemia. The aim of the present study was to delineate the area supplied by the inferior mesenteric artery with special consideration of the sigmoidea ima artery.

**Materials and Methods:**

The inferior mesenteric artery was dissected from its origin to the bifurcation of the superior rectal artery in 30 cadavers (15 male, 15 female). Vessel length and distance to the promontory were measured for each branch.

**Results:**

There were two manifestations of the sigmoidea ima artery, irrespective of the branching pattern of the inferior mesenteric artery. It originated below the promontory in 25 cases (83.3%) and above it in three (10%). It did not derive from the superior rectal artery in two cases (6.7%). In these 16.7%, the marginal artery was absent near the rectosigmoid junction.

**Conclusions:**

We suggest the terms “arteria sigmoidea ima pelvina” and “arteria sigmoidea ima abdominalis” for the two variants. The terms “arteria marginalis pelvina” and “arteria marginalis abdominalis” could be applied in clinical practice. An abdominal marginal artery could be considered a risk factor for colonic ischemia in colorectal resections and abdominal aortic aneurysm repair. Both variants should be considered when pre‐ and intra‐operative perfusion measurements are interpreted.

## 1 INTRODUCTION

The colorectal junction is a common site for the development of colonic ischemia. This is a clinical issue with nonocclusive ischemic colitis (Longo, Ballantyne, & Gusberg, 1992; Sun & Maykel, 2007; Trotter, Hunt, & Peter, 2016), colorectal resections (Park, Hur, Min, Lee, & Kim, 2012; Yamazaki et al., 1997,b; Yang, Lim, Yu, & Kim, 2016), and abdominal aortic aneurysm repair (Bjorck, Bergqvist, & Troeng, 1996). A number of studies have attempted to assess the blood supply to this area preoperatively (Khan, Goh, Tam, Wellsted, & Halligan, 2012) or intraoperatively (Jafari et al., 2015; Karliczek et al., 2010; Kudszus, Roesel, Schachtrupp, & Hoer, 2010; Watanabe et al., 2015) or to preserve perfusion by preserving the superior rectal artery (Borchert et al., 2015; Sohn et al., 2017; Tocchi et al., 2001). Watanabe et al. (2015) described cases of questionably sufficient or even missing blood flow along the colorectal junction using fluorescence angiography in vivo. In a cadaver study, van Tonder, Boon, Becker, and van Schoor (2007) found specimens with no anatomosis near the rectosigmoid junction. The aim of the present study was to look for an anatomical counterpart to the findings of Watanabe et al. (2015) and van Tonder et al. (2007) by detailed dissection of the inferior mesenteric artery with particular attention to the sigmoidea ima artery.

## 2 DEFINITIONS AND NOMENCLATURE

Anatomical terminology is used as follows: The bifurcation pattern of the inferior mesenteric artery (IMA) is distinguished by the form of its first branch. If its first branch provides no vessels before dividing into ascending and descending branches at the colonic border, it is named the left colic artery (LCA). If the first branch is a common stem for a sigmoid artery (SA) and the LCA, it is named the colosigmoid artery (CSA). After the first branch leaves the IMA, its continuation is named the superior rectal artery (SRA). On its way into the pelvis, the SRA branches to give a variable number of sigmoid arteries. The lowest sigmoid artery is named the sigmoidea ima (SI). The terminal branches of all the aforementioned vessels form the marginal artery near the colonic border. The terms “arteria colosigmoidea” (CSA) and “arteria sigmoidea ima” (SI) are taken from Manasse's publication. His definitions are adapted to make their applicability intuitive. The CSA is defined as originating from the IMA, dividing into the LCA plus SA and partially supplying the sigmoid. The SI is defined as the lowest sigmoid artery, which forms the distal ending of the marginal artery by linking with the SRA and partially providing blood to the sigmoid colon (Manasse, 1907).

## 3 MATERIALS AND METHODS

### 3.1 Dissection

**Figure 1:**
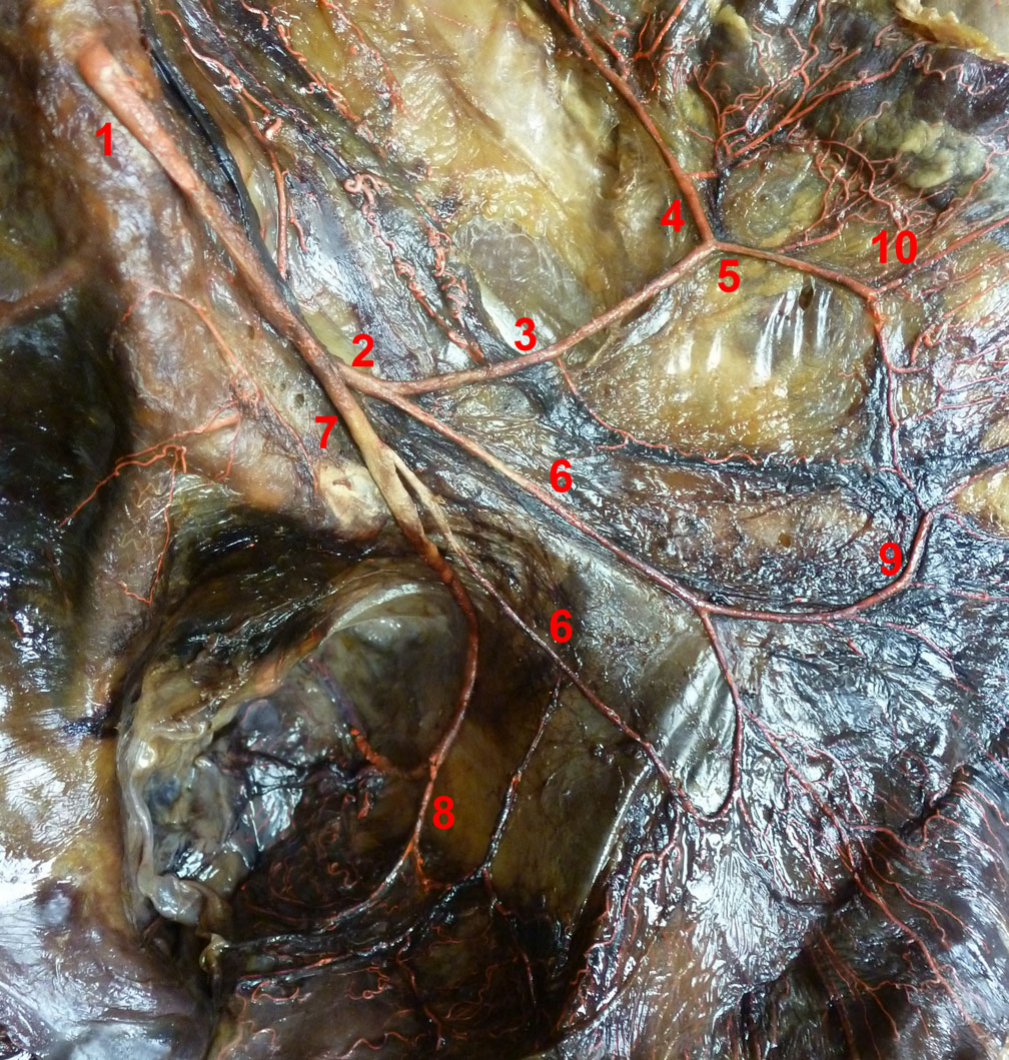
Example of a dissected specimen. Ventral view. 1: Inferior mesenteric artery originating from abdominal aorta, 2: Colosigmoid artery, 3: Left colic artery with ascending (4) and descending (5) branch, 6: Sigmoid arteries, 7: Superior rectal artery, 8: Sigmoidea ima artery, 9: Marginal artery, 10: Straight arteries [Color figure can be viewed at wileyonlinelibrary.com]

A total of 30 cadavers (15 male, 15 female) with no signs of surgery in the area supplied by the IMA were dissected. They were embalmed using the Thiel method (Thiel, 1992a) with the arteries filled with bright red latex solution for visibility (Thiel, 1992b). Dissection was started at the upper margin of the IMA and continued distally. The mesorectum was opened horizontally. Starting from its right edge, the incision was extended vertically toward the pelvic floor. All branches of the IMA and SRA were freed from fat and connective tissue as far as their entrance into the marginal artery. Dissection was stopped 2–3 cm distal to the SRA bifurcation (BIF; Figure 1).

### 3.2 Measurements

The length of an artery was measured from its origin to the proximal margin of its next branch. The upper margin of the promontory (PR) was used as reference point for measurements because it is an easily identified anatomical landmark. Parameters labeled “Length” were measured along the course of the respective vessel and those labeled “Distance” were measured as the shortest linear distance.

### 3.3 Statistical analysis

**Figure 2:**
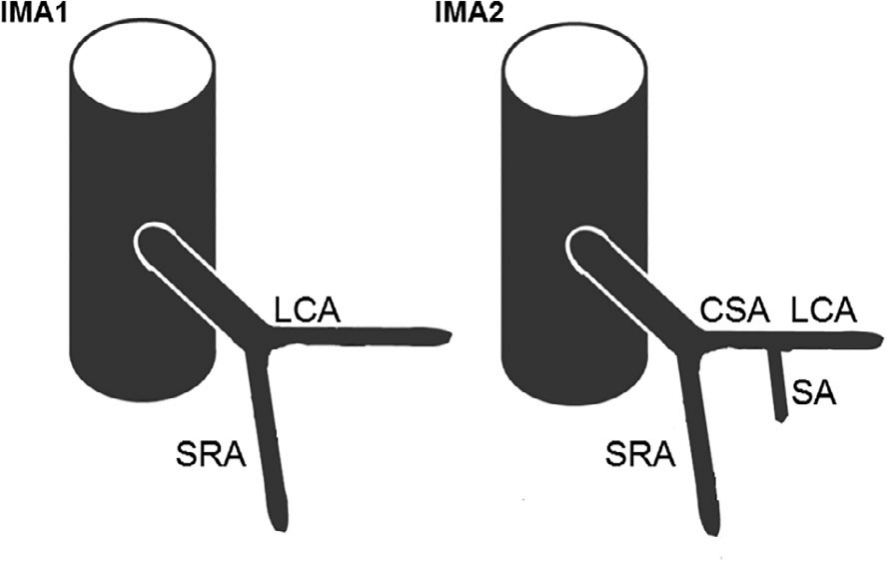
Differentiation of IMA branching patterns according to the form of the first branch. “IMA1” group is characterized by a left colic artery with no branches deriving. “IMA2” group is characterized by the colosigmoid artery, which forms a common stem for the LCA and a sigmoid artery *Source*: The illustration is adapted from Bertrand et al. (2014)

The sample was divided into two groups on the basis of the IMA branching pattern (Figure 2). The branching patterns were used as independent group variable and the measured parameters as dependent variables. Because of the small sample size, a Shapiro–Wilk test was used for the probability distribution. Differences in sample means were calculated using the *t*‐test or Mann–Whitney *U* test. The significance level was set at *α* = 0.05. In view of the nominal scaling of the group variable and the low numbers of some parameter measurements, Fisher's exact test (exact chi‐square test) with Freeman–Halton extension was used for contingency analysis. Correlations were measured using the contingency coefficient Cramérs V (*V*) and classified according to Cohen (Cohen's measurement of effect size).

## 4 RESULTS

### 4.1 Inferior mesenteric artery

Branching pattern IMA1 was observed in 16 cases and branching pattern IMA2 in 14. There was no significant difference in the length from the IMA origin to the LCA or CSA (*p* = .117). The length between the LCA or CSA and the promontory (*p* = .135) or the bifurcation of the SRA (*p* = .086) showed no significant differences. On average, the LCA left the IMA after 3.78 cm and the CSA after 4.33 cm. The SA branched off the CSA after 0.25–6.15 cm. The IMA1 group had significantly more branches deriving from the SRA than IMA2 (*p* = .007). The number of branches correlated significantly with the IMA branching pattern (*p* = .048). According to Cohen, this was a strong effect (*V* = 0.547). The number of branches located above the promontory showed a significant group difference (above: *p* = .006, below: *p* = .319) and a significant correlation with the IMA branching pattern (*p* = .01). This effect was strong (*V* = 0.563). There were no significant gender differences.

### 4.2 Superior rectal artery

In 22 of the 30 specimens, two or three branches originated from the SRA (73.3%). All IMA1 group cases showed at least one branch above and below the PR. In the IMA2 group, six had no branches above the PR and three had none below it. In one case, not a single branch originated from the SRA. The IMA1 group had one or two branches above the PR in 93.8% (one branch: 56.3%, two branches: 37.5%). The same pattern was found in 57.2% of the IMA2 group (one branch: 42.9%, two branches 14.3%). All measurements from the origin of the IMA and each branch of the SRA showed no significant group differences (Tables 1 and 2).

**Table 1:** Measurements regarding the first branch of the groups IMA1 and IMA2

	IMA1				IMA2			
	Min	Max	*M*	*s* ^2^	Min	Max	*M*	*s* ^2^
Length: IMA → LCA/CSA	2.10	5.95	3.78	1.16	3.25	5.70	4.33	0.55
Length: LCA/CSA	0.25	12.75	6.69	17.56	0.25	6.15	1.31	3.87
Length: LCA/CSA → PR	3.75	11.70	7.47	4.48	2.20	9.20	6.24	5.17
Length: LCA/CSA → BIF	6.80	14.60	11.31	4.10	4.95	14.05	9.72	8.19

*Note*: Parameters in cm.

**Table 2:** Measurements regarding the IMA and branches of the SRA

	Measurements	IMA1				IMA2			
	*N* (IMA1, IMA2)	Min	Max	*M*	*s* ^2^	Min	Max	*M*	*s* ^2^
Distance: IMA → PR	30 (16, 14)	6.20	12.00	9.58	3.35	5.75	13.30	9.20	4.18
Distance: LCA/CSA → PR	30 (16, 14)	2.50	9.55	6.29	3.62	1.35	8.25	5.26	4.57
Distance: B1 → PR	29 (16, 13)	1.45	7.60	4.17	3.00	−2.50	7.30	2.64	9.84
Distance: B2 → PR	25 (16, 9)	−3.65	4.15	−0.14	4.56	−4.10	2.05	−1.63	3.83
Distance: B3 → PR	12 (9, 3)	−2.95	0.60	−1.57	1.30	−2.75	−1.40	−1.98	0.48
Distance: B4 → PR	3 (3, 0)	−5.00	−1.50	−2.78	3.72	–	–	–	–
Distance: BIF → PR	30 (16, 14)	−8.20	1.10	−2.93	4.79	−5.35	0.80	−2.70	1.95

*Note*: Parameters in cm. B1 = first branch of the SRA, B2 = second branch, B3 = third branch, B4 = fourth branch.

### 4.3 Sigmoidea ima artery

**Figure 3:**
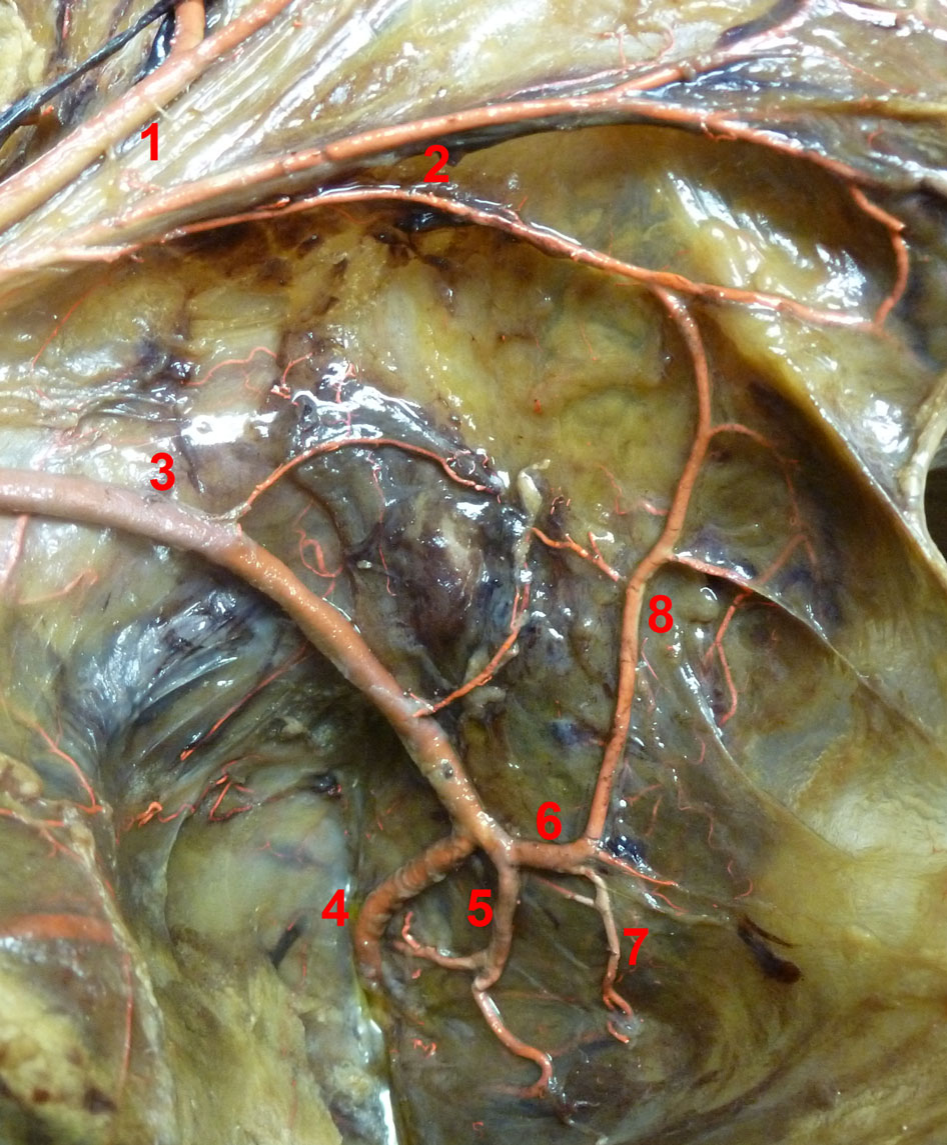
Sigmoidea ima artery originating from the SRA bifurcation. View from ventrolateral right. 1: Colosigmoid artery, 2: Sigmoid arteries, 3: Superior rectal artery, 4/5: Left/Right branch of SRA bifurcation, 6: Sigmoidea ima artery, 7: Descending branch of sigmoidea ima artery, 8: Marginal artery [Color figure can be viewed at wileyonlinelibrary.com]

**Figure 4:**
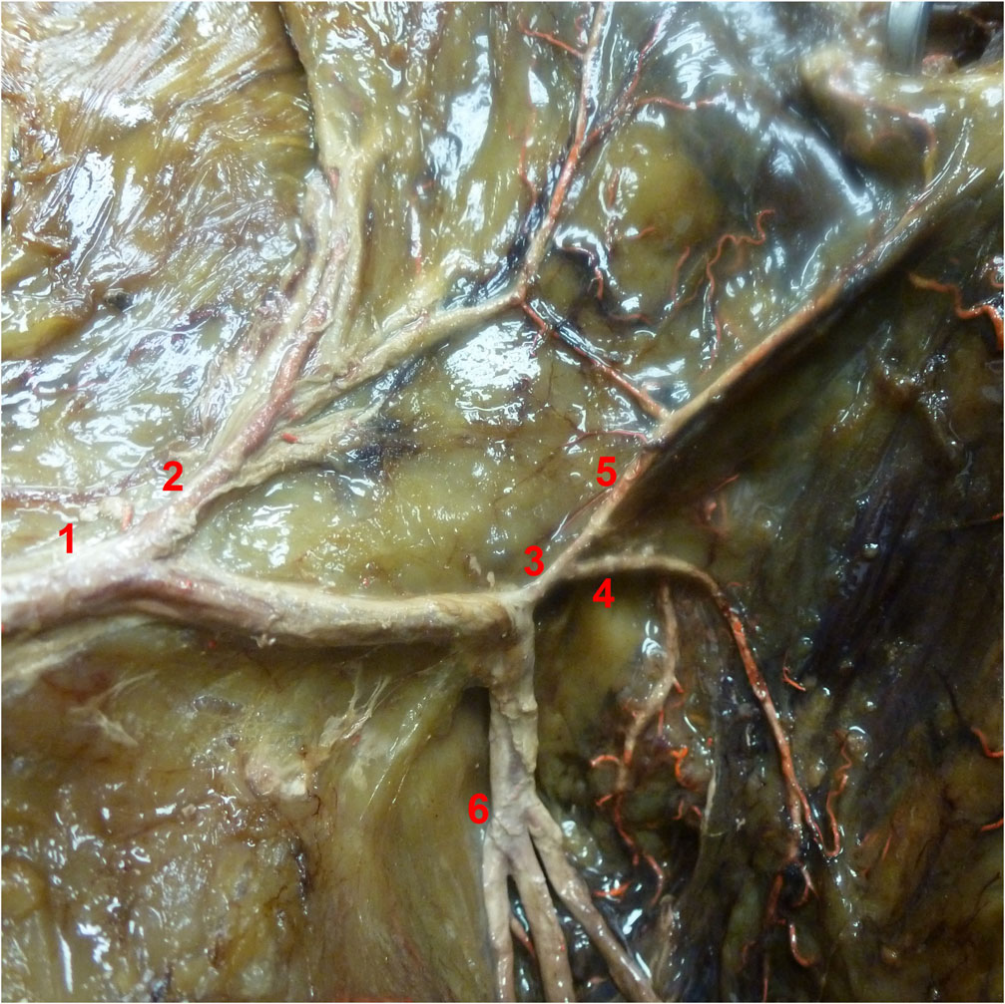
Sigmoidea ima artery originating from the SRA. View from ventrolateral right. 1: Superior rectal artery, 2: Sigmoid artery, 3: Sigmoidea ima artery with descending branch (4), 5: Marginal artery, 6: Trifurcation of superior rectal artery into right, left and posterior branches [Color figure can be viewed at wileyonlinelibrary.com]

In five specimens, the lowest sigmoid artery did not form the distal end of the marginal artery and Manasse's definition could not be applied. In these cases, the next most proximal vessel, which formed the ending of the marginal artery, was considered as the SI. The groups differed significantly in the branching number of the SI (*p* = .002). The term “branching number” describes the position of the SI origin from the SRA with its branches numbered in ascending order from proximal to distal. The branching number correlated significantly with the branching pattern of the IMA (*p* = .017). This effect was strong (*V* = 0.591). In 16 cases, the SI left the SRA as second branch (IMA1: 50%, IMA2: 57.1%). In other cases, it preferentially took higher branching numbers in the IMA1 group and lower ones in IMA2. The SI originated below the promontory in 25 cases (83.3%) and above it in three (10%). In two cases, it did not derive from the SRA (6.7%). In both of those, the next proximal branch was the CSA (origin “CSA” in Table 3). In these 16.7%, the marginal artery was absent near the colorectal junction. However, in all three specimens where the SI originated above the PR, a long descending branch derived from the SI and headed toward the rectosigmoid junction. There were no group differences in terms of the origin of the SI, its position relative to the PR, its proximal end or its length. There were no significant gender differences in any of the measurements. Tables 3, 4, 5 display all measurements relating to the SI. Figures 3 and 4 illustrate the SI artery in two dissected specimens.

**Table 3:** Observations regarding the sigmoidea ima artery

			Origin				Location to the PR		End	
	*B*	*N*	CSA	SRA	Rdex	Rsin	Above	Below	SA	Rdesc
IMA1	2	8	–	5	–	3	1	7	5	3
	3	6	–	4	–	2	–	6	5	1
	4	2	–	–	–	2	–	2	2	–
IMA2	0	2	2	–	–	–	–	–	–	–
	1	3	–	3	–	–	1	2	2	1
	2	8	–	5	1	2	1	7	4	4
	3	1	–	1	–	–	–	1	1	–

*Note*: B = branching number, Rdex = right branch of the SRA bifurcation, Rsin = left branch, Rdesc = descending branch originating from the sigmoidea ima.

**Table 4:** Distances between the origin of sigmoidea ima artery and the promontory

	Measurements	Parameters			
	*N*	Min	Max	*M*	*s* ^2^
IMA1					
Distance: SI (B2) → PR	8	−3.65	0.25	−1.58	1.79
Distance: SI (B3) → PR	6	−2.95	−0.80	−2.09	0.72
Distance: SI (B4) → PR	2	−1.85	−1.50	−1.68	0.06
IMA2					
Distance: SI (B1) → PR	3	−1.95	2.25	−0.08	4.57
Distance: SI (B2) → PR	8	−4.10	0.70	−2.09	2.20
Distance: SI (B3) → PR	1	−1.40	−1.40	−1.40	–
IMA1 + 2					
Distance: SI → PR	28	−4.10	2.25	−1.68	1.88
Distance: SI below PR	25	−4.10	−0.20	−2.01	0.97
Distance: SI above PR	3	0.25	2.25	1.07	1.10

*Note*: Parameters in cm. B1 = first branch of the SRA, B2 = second branch, B3 = third branch, B4 = fourth branch.

**Table 5:** Measurements regarding the sigmoidea ima artery

	Measurements	IMA1				IMA2			
	*N* (IMA1, IMA2)	Min	Max	*M*	*s* ^2^	Min	Max	*M*	*s* ^2^
Length: IMA → SI	28 (16, 12)	8.85	15.80	13.75	3.08	5.60	16.65	12.83	7.05
Length: LCA/CSA → SI	28 (16, 12)	3.95	12.10	9.97	3.78	1.70	13.40	8.51	7.50
Length: SRA → SI (B1)	3 (0, 3)	–	–	–	–	1.70	9.00	6.48	17.18
Length: SRA → SI (B2)	16 (8, 8)	3.95	12.00	9.46	6.43	6.65	13.40	9.02	4.18
Length: SRA → SI (B3)	7 (6, 1)	9.35	11.50	10.30	0.88	10.50	10.50	10.50	–
Length: SRA → SI (B4)	2 (2, 0)	9.95	12.10	11.03	2.31	–	–	–	–
Length: PR → SI									
Location above the PR	3 (1, 2)	0.45	0.45	0.45	–	1.10	5.60	3.35	10.13
Location below the PR	25 (15, 10)	0.20	5.30	2.69	2.11	0.50	4.45	2.76	1.91
Length: SI → BIF									
Location above the BIF	18 (9, 9)	0.30	10.65	3.23	9.61	0.70	6.50	2.88	4.38
Location below the BIF	10 (7, 3)	0.10	4.70	1.09	2.61	0.55	1.40	0.85	0.23
Length: SI	28 (16, 12)	0.50	16.55	5.23	17.58	0.80	11.05	4.18	14.02
Length: SI (B1)	3 (0, 3)	–	–	–	–	2.80	9.55	7.08	13.87
Length: SI (B2)	16 (8, 8)	0.50	16.55	6.41	29.36	0.80	11.05	3.48	12.46
Length: SI (B3)	7 (6, 1)	0.65	6.90	4.08	5.98	1.00	1.00	1.00	–
Length: SI (B4)	2 (2, 0)	2.25	5.75	4.00	6.13	–	–	–	–
Length: SI (SA)	19 (12, 7)	2.20	16.55	6.26	17.60	1.00	11.05	6.11	14.77
Length: SI (Rdesc)	9 (4, 5)	0.50	5.90	2.15	6.47	0.80	2.80	1.46	0.61

*Note*: Parameters in cm. SA = SI ended at next proximal sigmoid artery, Rdesc = SI ended at descending branch.

## 5 DISCUSSION

### 5.1 Inferior mesenteric artery

To allow direct comparisons to be made, the works of Adachi (1928), Sierocinski (1976), Lippert and Pabst (1985), Nelson, Raymond, Olga, and Herand (1988), Bertrand et al. (2014) and Murono et al. (2015) were adapted to the branching classification used in this study (Table 6). Despite similar results by Adachi (1928) and Bertrand et al. (2014), the data compared are inconsistent and no conclusive evaluation of the distribution of the branching classification chosen in this study is possible. The lengths between the IMA origin and its first branch are similar to the data found in the literature (Table 7).

**Table 6:** Frequency distribution of IMA branching patterns

		IMA1		IMA2		IMAX	
Authors, date of publication	Study design	*N*	%	*N*	%	*N*	%
Adachi (1928)	Dissection	34	52.3	31	47.7	–	–
Sierocinski (1976)	Dissection	88	88	10	10	2	2
Lippert and Pabst (1985)	Meta‐analysis	–	30.0	–	64.0	–	6.0
Nelson et al. (1988)	Dissection	14	28.0	33	66.0	3	6.0
Bertrand et al. (2014)	CT angiography	44	51.2	42	48.8	–	–
Murono et al. (2015)	CT angiography	193	41.2	251	53.7	24	5.1
Own results (2019)	Dissection	16	53.3	14	46.7	–	–

*Note*: The data of Adachi, Sierocinski, Lippert and Pabst, Nelson et al., Bertrand et al., and Murono et al. were adapted to the branching classification shown in Figure 2. IMAX = branching pattern could not be adapted.

**Table 7:** Comparison of lengths between IMA origin and its first branch

		Length: IMA → LCA/CSA			
Authors, date of publication	Study design	*N*	Min	Max	*M*
Michels, Siddharth, Kornblith, and Parke (1965)	Dissection	127	2.0	7.0	3.5
Zebrowski, Augustyniak, and Zajac (1971)	Dissection	115	1.0	7.0	–
Sierocinski (1976)	Dissection	100	1.2	5.4	–
Bergman (1988)	Dissection	115	1.0	7.0	–
Murono et al. (2015)	CT angiography	471	1.1	8.2	3.9
Own results (2019)	Dissection	30	2.1	6.0	3.8

*Note*: Parameters in cm.

### 5.2 Sigmoidea ima artery

**Figure 5:**
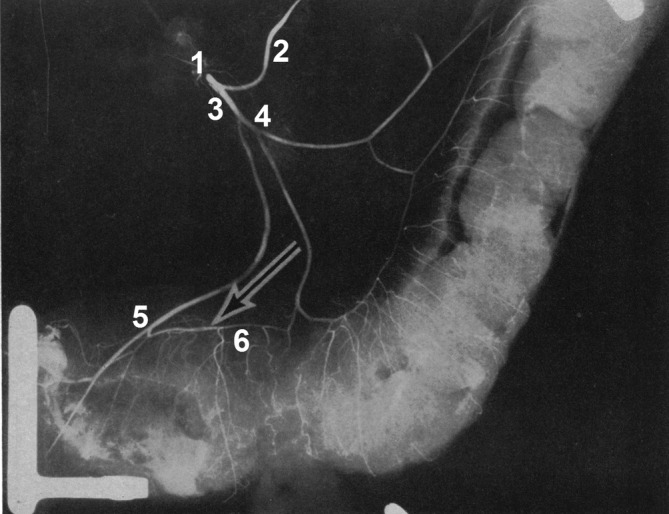
Inferior mesenteric artery (1) branches into left colic artery (2) and superior rectal artery (3). A sigmoid artery (4) branches off before the sigmoidea ima artery (5) leaves the SRA and therewith ends the marginal artery (6) *Source*: Illustration of an injected specimen taken from Griffiths (1961)

No data were found to compare SI branching numbers. Interestingly, Griffiths (1961) included an illustration of an injected specimen in which the arterial branching is similar to findings in the present article (Figure 5). Multiple studies have described the location of the SI. Manasse (1907) and Sunderland (1942) located it “deep in the sacral hollow”. Rubesch (1910), Pope and Buie (1929) and VanDamme, Bonte, and van der Schueren (1982) quantified the location as 1–2 cm below the promontory, consistent with the findings in the current article. This work describes three cases with the SI originating above the PR and two with no direct connection between the SI and SRA. These observations are consistent with Manasse (1907) and Sunderland (1942), both of whom found SIs not contributing to the marginal artery at the rectosigmoid junction. However, this pattern is not common. Sunderland (1942), Greenberg (1950) (81%), van Tonder et al. (2007) (95.3%), and Sierocinski (1976) (100%) consistently found an anastomosis between the SI and the SRA. In contrast, Michels et al. (1965) (52%) only found it in around half of their cases. Cases with the SI deriving below the bifurcation of the SRA have been reported. Sunderland (1942) noticed it in 20% and Greenberg (1950) in 5.4%. A descending branch was less common in the present article (30%) than reported by VanDamme et al. (1982) (65%).

In the present study, SI length ranged from 0.5 to 16.55 cm. Manasse (1907) described the SI as a very short vessel. VanDamme et al. (1982) measured it from 0.5 to 1.0 cm. Sunderland (1942) stated without quantification that it could be a very large descending vessel. The one specimen with no branches leaving the SRA described in the present article is rare but not novel. VanDamme and Bonte (1990) and Bertrand et al. (2014) also reported it.

### 5.3 Correlation between IMA branching pattern and nature of the sigmoidea ima artery

Embryology could explain the correlation between the IMA branching pattern and the number of SRA branches or SI branching number. Lin and Chaikof (2000) reported that the number and form of sigmoid arteries depend on the character of the sigmoid mesocolon. If the base of the mesosigmoid is narrow, the sigmoid arteries tend to originate from the SRA (IMA1 group in this article). If the base of the mesosigmoid is wide, the sigmoid arteries tend to originate from a common stem (IMA2 group).

### 5.4 Defining and naming the sigmoidea ima artery—A suggestion

We suggest a definition and nomenclature for the sigmoidea ima artery based on anatomical, clinical, and historical data. Historically, the SI artery has been closely linked to the controversy surrounding Sudeck's critical point. In 1907, Paul Sudeck identified a critical point in the arterial supply to the colon and rectum using injection experiments. He located the critical point at the entrance of the last sigmoid artery into the SRA (Sudeck, 1907). Subsequently, the lowest sigmoid artery was termed “arteria sigmoidea ima” (Manasse, 1907). The sigmoidea ima was defined hierarchically. As the name‐giver, Manasse's publication gained the highest priority; subsequent aspects were prioritized according to their authority in the literature, including the results in this article.

1. The sigmoidea ima is the lowest sigmoid artery (Latin “ima” = “lowest”) (Manasse, 1907).
2. The sigmoidea ima forms the distal ending of the marginal artery by linking with the SRA (Manasse, 1907).
3. The sigmoidea ima mostly occurs:

- in the pelvic cavity, 1–2 cm below the promontory (Manasse, 1907; Rubesch, 1910; Pope & Buie, 1929; Sunderland, 1942; VanDamme et al., 1982; present results);
- with an anastomosis to the SRA, which is located above its bifurcation (Sunderland, 1942; Greenberg, 1950; Sierocinski, 1976; van Tonder et al., 2007; present results);
- or below its bifurcation (Rubesch, 1910; Sunderland, 1942; Greenberg, 1950; present results).

4. The sigmoidea ima rarely occurs in the abdominal cavity, that is, above the promontory (Manasse, 1907; Sunderland, 1942; present results).

**Figure 6:**
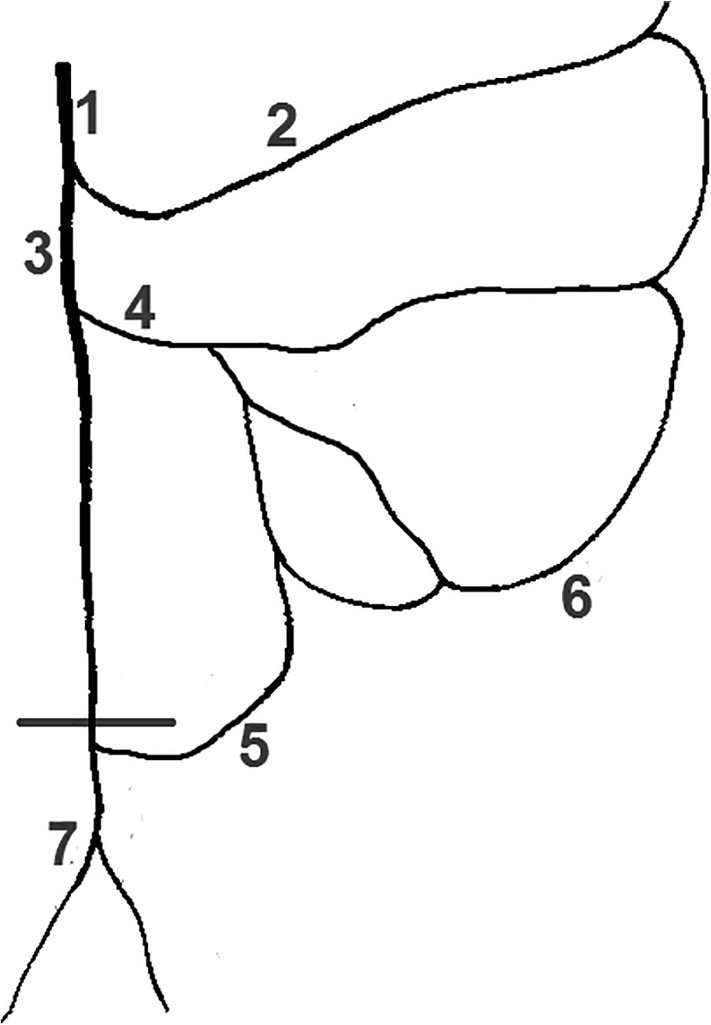
Sketch of the colorectal perfusion. Inferior mesenteric artery (1) divides into left colic artery (2) and superior rectal artery (3). The SRA first branches off a sigmoid artery (4) above and a pelvic sigmoidea ima artery (5) below the promontory (horizontal line). The sigmoidea ima marks the last vessel of the marginal artery (6). The SRA bifurcates (7) into right and left branches. This pattern was found in five cases in the IMA1 and IMA2 groups *Source*: Sketch adapted from Sunderland (1942)

**Figure 7:**
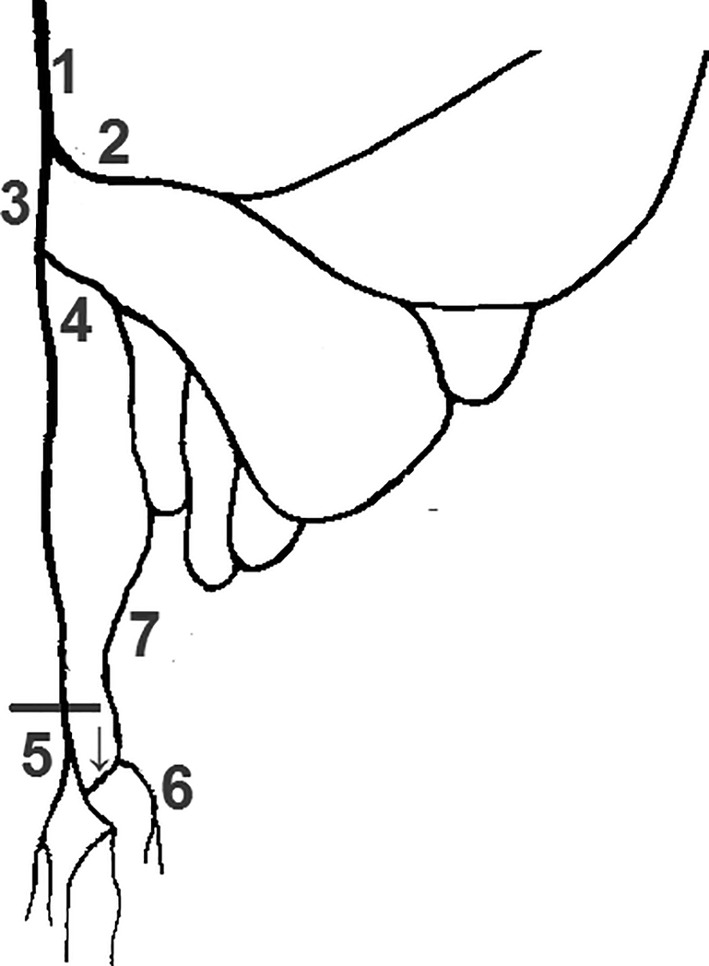
Sketch of the colorectal perfusion. Inferior mesenteric artery (1) divides into colosigmoid artery (2) and superior rectal artery (3). A sigmoid artery (4) originates before a pelvic sigmoidea ima artery (arrow) leaves the SRA as second branch. Its origin is located below the promontory (horizontal line) and below the bifurcation of the SRA (5). The SI branches off a descending branch (6). (7) Denotes the marginal artery. This pattern was found three times in each group (IMA1 and 2) *Source*: Sketch adapted from Sunderland (1942)

**Figure 8:**
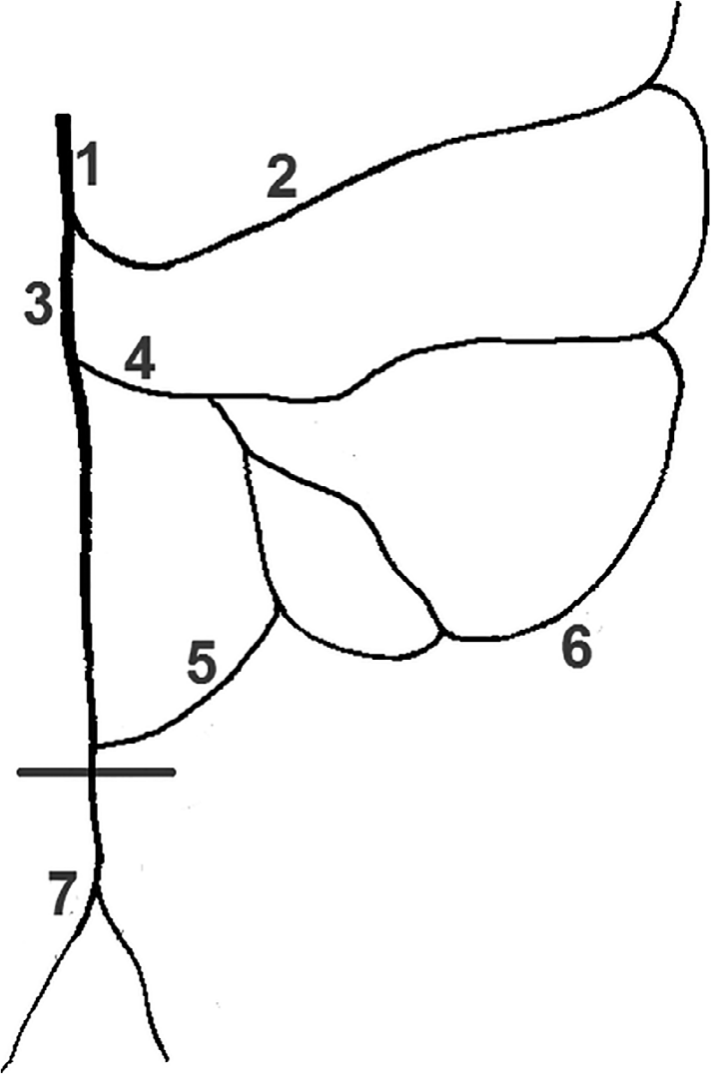
Sketch of the colorectal perfusion. An abdominal sigmoidea ima artery (5) branches off above the promontory (horizontal line). This pattern was found in three specimens. 1: Inferior mesenteric artery, 2: Left colic artery, 3: Superior rectal artery, 4: Sigmoid artery, 5: Sigmoidea ima abdominalis, 6: Marginal artery, 7: Bifurcation of SRA *Source*: Sketch adapted from Sunderland (1942)

The decisive characteristic of both manifestations is their location relative to the PR. In order to distinguish the two manifestations while including their relationship to the PR, the terms “arteria sigmoidea ima pelvina” and “arteria sigmoidea ima abdominalis” could be applied. This nomenclature is motivated by the existing anatomical border between the abdominal and pelvic cavities, with the PR conveniently being part of it. Figures 6, 7, 8 illustrate the newly introduced nomenclature in sketches.

### 5.5 Nomenclature in clinical practice

**Figure 9:**
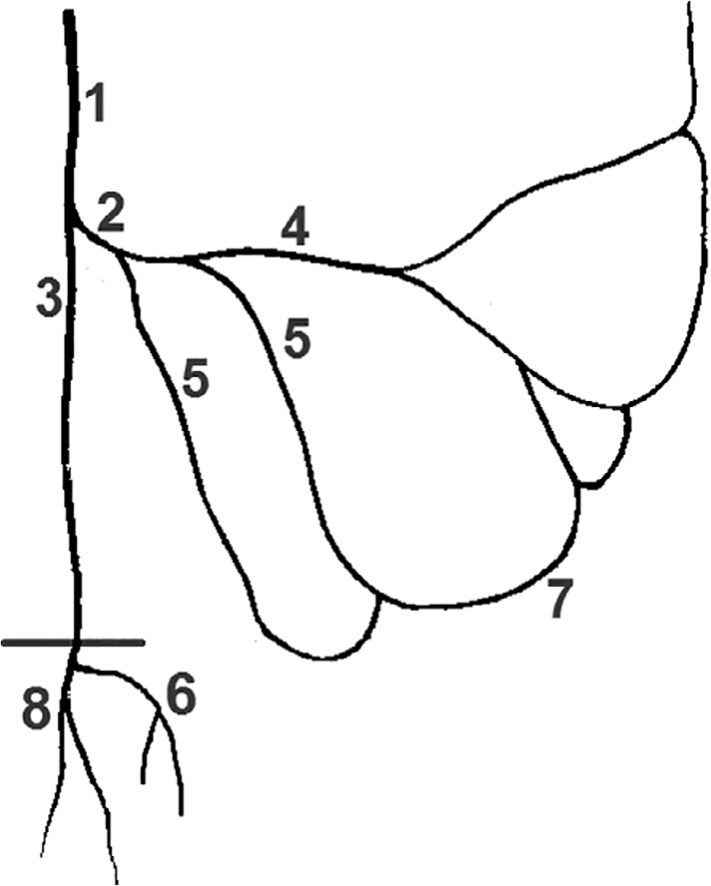
Sketch of the colorectal perfusion. The distal ending of the marginal artery is not formed by the lowest sigmoid artery (6) but rather the colosigmoid artery (2). The pattern shown was found in two cases of group IMA2. 1: Inferior mesenteric artery, 3: Superior rectal artery, 4: Left colic artery, 5: Sigmoid arteries, 7: Marginal artery, 8: Bifurcation of SRA. Horizontal line: Promontory *Source*: Sketch adapted from Sunderland (1942)

In rare cases, the lowest sigmoid artery does not form the distal ending of the marginal artery (Figure 9) because there is no anastomosis with the next proximal vessel or no sigmoid arteries deriving from the SRA. In these cases, the etymology of the sigmoidea ima (“ima” = “lowest” in Latin) and the reason for its invention to denote the distal end of the marginal artery explicitly do not match logically. The crucial clinical aspect is the presence or absence of a sufficient marginal artery at the colorectal junction, regardless of which vessel actually forms its distal ending. Accordingly, we suggest the terms pelvic marginal artery (“arteria marginalis pelvina”) and abdominal marginal artery (“arteria marginalis abdominalis”) for clinical practice.

### 5.6 Clinical relevance

High tying of the IMA is reported to reduce blood flow in the marginal artery‐dependent sigmoid colon (Dworkin & Allen‐Mersh, 1996). The combination of reduced blood flow in the oral‐side marginal artery and the presence of an abdominal marginal artery, which could be interpreted as impaired blood flow from the anal‐side marginal artery, could endanger the supply to the rectosigmoid. This could explain colonic ischemia after colorectal resections (Park et al., 2012; Yamazaki et al., 1997,b; Yang et al., 2016) and abdominal aortic aneurysm repair (Bjorck et al., 1996). The absence of a pelvic marginal artery could explain the results of Watanabe et al. (2015), who, using intraoperative fluorescence angiography with indocyanine green, saw cases of missing perfusion at the rectosigmoid junction. In such cases, the marginal artery could end above the pelvic brim as an abdominal marginal artery. Awareness of the two different ending locations of the marginal artery could be taken into account for future pre‐ and intra‐operative perfusion studies.

### 5.7 Limitations

The limitations of the present study are the sample size and the sometimes difficult comparisons between our results and the literature owing to differing definitions and nomenclatures. Also, a cadaver study is based on the premise that lengths, locations and diameters are similar in vivo and postmortem.

## 6 CONCLUSIONS

Our dissection study revealed two manifestations of the sigmoidea ima artery. In most cases, the SI originated below the promontory at an average of 2 cm. In three cases, it was located above the pelvic brim. We suggest the terms “arteria sigmoidea ima pelvina” and “arteria sigmoidea ima abdominalis” to distinguish these variants. Neither the branching pattern of the IMA nor the number of branches deriving from the SRA significantly affected the location of the SI. In five specimens, the marginal artery was absent at the rectosigmoid junction, in three cases because the SI was abdominal and in two because there was no direct link between the sigmoid artery and SRA. Consequently, the terms “arteria marginalis pelvina” and “arteria marginalis abdominalis” could be more useful in clinical practice. The presence of an abdominal marginal artery could be considered a risk factor for colonic ischemia in colorectal resections and abdominal aortic aneurysm repair. Our results should also be considered when pre‐ and intra‐operative perfusion measurements are interpreted.
